# Exendin-4 and sitagliptin protect kidney from ischemia-reperfusion injury through suppressing oxidative stress and inflammatory reaction

**DOI:** 10.1186/1479-5876-11-270

**Published:** 2013-10-25

**Authors:** Yen-Ta Chen, Tzu-Hsien Tsai, Chih-Chau Yang, Cheuk-Kwan Sun, Li-Teh Chang, Hung-Hwa Chen, Chia-Lo Chang, Pei-Hsun Sung, Yen-Yi Zhen, Steve Leu, Hsueh-Wen Chang, Yung-Lung Chen, Hon-Kan Yip

**Affiliations:** 1 Division of Urology, Kaohsiung Chang Gung Memorial Hospital and Chang Gung University College of Medicine, Kaohsiung, Taiwan; 2 Division of Cardiology, Department of Internal Medicine, Kaohsiung Chang Gung Memorial Hospital and Chang Gung University College of Medicine, 123, Dapi Road, Niaosung Dist., Kaohsiung city 83301, Taiwan; 3 Division of Nephrology, Department of Internal Medicine, Kaohsiung Chang Gung Memorial Hospital and Chang Gung University College of Medicine, Kaohsiung, Taiwan; 4 Department of Emergency Medicine, E-DA Hospital, I-Shou University, Kaohsiung, Taiwan; 5 Basic Science, Nursing Department, Meiho University, Pingtung, Taiwan; 6 Division of Colorectal Surgery, Department of Surgery, Kaohsiung Chang Gung Memorial Hospital and Chang Gung University College of Medicine, Kaohsiung, Taiwan; 7 Center for Translational Research in Biomedical Sciences, Kaohsiung Chang Gung Memorial Hospital and Chang Gung University College of Medicine, Kaohsiung, Taiwan; 8 Department of Biological Sciences, National Sun Yat-Sen University, Kaohsiung, Taiwan

**Keywords:** Exendin-4, Sitagliptin, Acute ischemia-reperfusion injury, Inflammation, Oxidative stress

## Abstract

**Background:**

This study tested the hypothesis that exendin-4 and sitagliptin can effectively protect kidney from acute ischemia-reperfusion (IR) injury.

**Methods:**

Adult SD-rats (n = 48) equally divided into group 1 (sham control), group 2 (IR injury), group 3 [IR + sitagliptin 600 mg/kg at post-IR 1, 24, 48 hr)], and group 4 [IR + exendin-4 10 μm/kg at 1 hr after procedure] were sacrificed after 24 and 72 hrs (n = 6 at each time from each group) following clamping of bilateral renal pedicles for 60 minutes (groups 2–4).

**Results:**

Serum creatinine level and urine protein to creatinine ratio were highest in group 2 and lowest in group 1 (all p < 0.001) without notable differences between groups 3 and 4. Kidney injury score, expressions of inflammatory biomarkers at mRNA (MMP-9, TNF-α, IL-1β, PAI-1), protein (TNF-α, NF-κB and VCAM-1), and cellular (CD68+) levels in injured kidneys at 24 and 72 hr showed an identical pattern compared to that of creatinine level in all groups (all p < 0.0001). Expressions of oxidized protein, reactive oxygen species (NOX-1, NOX-2), apoptosis (Bax, caspase-3 and PARP), and DNA damage marker (γH2AX+) of IR kidney at 24 and 72 hrs exhibited a pattern similar to that of inflammatory mediators among all groups (all p < 0.01). Renal expression of glucagon-like peptide-1 receptor, and anti-oxidant biomarkers at cellular (GPx, GR) and protein (NQO-1, HO-1, GPx) levels at 24 and 72 hr were lowest in group 1, significantly lower in group 2 than in groups 3 and 4 (all p < 0.01).

**Conclusion:**

Exendin-4 and sitagliptin provided significant protection for the kidneys against acute IR injury.

## Background

Acute kidney injury (AKI) is a commonly encountered complication in hospitalized patients and significantly contributes to morbidity and mortality [1-5]. Recent studies have further demonstrated that AKI was evident in around 20% of patients who died in hospitals and up to 50% of patients in the intensive care unit (ICU) [6,7]. The etiology of AKI is multifactorial [1-12]. Among the various etiologies of hospital-acquired AKI, ischemia-reperfusion (IR) injury is the leading cause of AKI [13-15] that is associated with a high mortality rate [16,17]. The causes of acute kidney IR injury are divergent, including contrast media-induced nephropathy [9,18], shock followed by resuscitation in the emergency and intensive care settings [11,19], kidney transplantation [20,21], sepsis [22], and cardiovascular surgery [23].

Previous studies [11,15,23-27] have reported that the underlying mechanisms of acute kidney IR injury are mainly through the generation of oxidative stress and reactive oxygen species (ROS), rigorous inflammatory reaction, and enhancement of cellular apoptosis after prolonged or even transient IR injury [13,22,28]. Experimental studies have further revealed that inhibition of inflammatory reaction and suppression of the generations of pro-inflammatory cytokines and oxidative stress using immuno- or pharmaco-modulation significantly protect the kidney from acute IR injury [23-27].

Glucagon-like peptide-1 (GLP-1)-based pharmaceuticals are emerging as potent regimens against type 2 diabetes mellitus (T2DM). Exendin-4 and liraglutide, two GLP-1 analogues, have been reported to have multiple cellular protective effects, including the protection of endothelial cells against senescence mainly through anti-oxidative [29-31] and anti-inflammatory [31-33] processes. Additionally, studies have revealed that GLP-1 mediates in the therapeutic actions of dipeptidyl peptidase (DPP)-IV inhibitors [34]. Interestingly, sitagliptin, currently used for treating type 2 diabetic patients, has been found to be able to enhance circulating GLP-1 levels through inhibition of DPP-IV activity [35,36] which, in turn, provides cardiovascular protective effect probably through the anti-inflammatory and anti-atherosclerotic actions of GLP-1 [37]. Thus, it is rational to hypothesize that the inflammatory reaction and oxidative stress from acute renal IR injury may be alleviated by either Exendin-4 (i.e., GLP-1 analogue) or sitagliptin treatment through the induction of GLP-1 receptor (GLP-1R) expression.

## Materials and methods

### Ethics

All animal experimental procedures were approved by the Institute of Animal Care and Use Committee at Kaohsiung Chang Gung Memorial Hospital (Affidavit of Approval of Animal Use Protocol No. 2008121108) and performed in accordance with the Guide for the Care and Use of Laboratory Animals (NIH publication No. 85–23, National Academy Press, Washington, DC, USA, revised 1996).

### Animal grouping and induction of acute kidney ischemia-reperfusion injury

Pathogen-free, adult male Sprague–Dawley (SD) rats (n = 48) weighing 320–350 g (Charles River Technology, BioLASCO Taiwan Co. Ltd., Taiwan) were randomized and equally divided into group 1 (sham controls, n = 12), group 2 (acute kidney IR injury only, n = 12), group 3 (sitagliptin 600 mg/kg orally at post-IR 1, 24, and 48 hr), and group 4 (IR + sitagliptin 600 mg/kg orally + exendin-4 10 μm/kg subcutaneous injection at post-IR 1 hr). The rats were sacrificed at post-IR 24 hr (3 hrs after sitagliptin treatment) and 72 hr (n = 6 each time for each group) for determining the therapeutic effects of sitagliptin and exendin-4 at acute (i.e., 24 hr) and subacute (i.e., 72 hr) phases of IR injury.

All animals were anesthetized by inhalational 2.0% isoflurane, placed supine on a warming pad at 37°C for midline laparotomies. Sham-operated rats (group 1) received laparotomy only, while acute IR injury of both kidneys were induced in all animals in groups 2 to 4 by clamping the renal pedicles for one hour using non-traumatic vascular clips. The rats were sacrificed at 24 and 72 hrs (n = 6 at each time from each group) after IR procedure. The kidneys were harvested for individual study.

### Rationale of drug dosage for the study

To elucidate relatively suitable drug dosages for the present study, acute kidney IR injury in four additional rats was treated by either a low (n = 2) or a high (n = 2) dose of sitagliptin (i.e., 200 or 600 mg/kg/day for three days, respectively). Similarly, four other rats were treated with either a low (n = 2) or a high (n = 2) dose of exendin-4 six (i.e., 5 or 10 μg/kg once, respectively) after renal IR induction. Immunohistochemical (IHC) staining and the protein expressions of GLP-1R in kidney parenchyma were notably higher in the rats treated with a high dose of sitagliptin or exendin-4 compared with those receiving low doses of the two drugs. Thus, 600 mg/kg/day of sitagliptin for three successive days and 10 μg/kg (single dose only) of exendin-4 were utilized in the current study.

**Figure 1:**
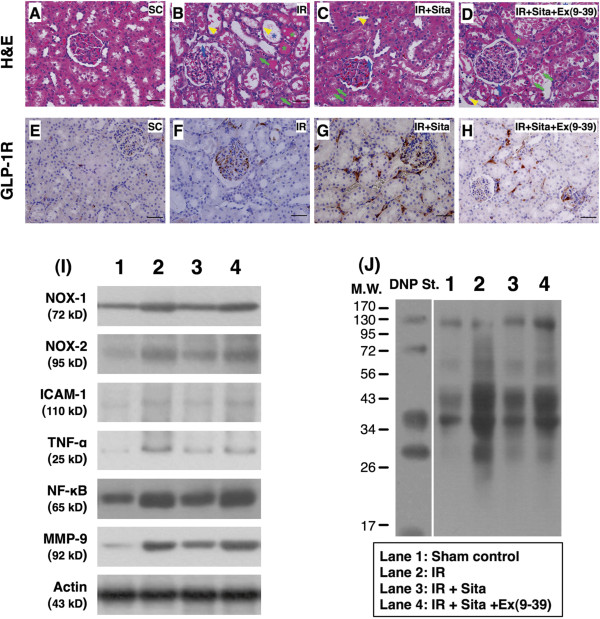
**Immunohistochemical (IHC) and H.E. staining and Western blot for determining the impact of sitagliptin and exendin-9-39 effect on acute kidney ischemia-reperfusion (IR) injury at 24 h after IR procedure. A** to **D)** The H.E stain (200 x) showing notably higher acute kidney injury score, including: brush border in renal tubules (yellow arrowheads), cast formation (green asterisk), tubular dilatation (blue asterisk), tubular necrosis (green arrows), and dilatation of Bowman’s capsule (blue arrows) in ischemia reperfusion (IR) only and IR + sitagliptin (Sita) + exendin (Ex)-9-39 animals than in IR + sitagliptin animals. **E** to **H)** The immunohistochemical (IHC) stain (200 x) demonstrating higher expression of glucagon-like peptide-1 receptor (GLP-1R) (brown color staining) in IR animals with sitagliptin + exendin-9-39 treatment and further higher expression of this biomarker than in RI only animals in renal parenchyma. Very few expression of GLP-1R in normal kidney parenchyma **(E)** was observed. Scale bars in right lower corner represent 50 μm. **I)** The protein expressions of ADPH oxidase (NOX)-1, NOX-2, intercellular adhesion molecule (ICAM)-1, tumor necrotic factor (TNF)-α, nuclear factor (NF)- κB, matrix metalloproteinase (MMP)-9, and **J)** oxidized protein were remarkably higher in IR and IR + sitagliptin + exendin-9-39 animals than in IR + sitagliptin animals.

To elucidate the possible GLP-1-mediated therapeutic effect of sitagliptin against acute kidney IR injury, the circulating level of GLP-1 was measured in each animal. In addition, eight additional SD rats were equally divided into: 1) sham control, 2) IR only, 3) IR + sitagliptin 600 mg/kg (orally, 1 hr after acute kidney IR), 4) IR + sitagliptin 600 mg/kg + exendin-9-39 (an inhibitor of exendin-4) 10 μm/kg at 1 hr after the procedure. The animals were sacrificed at 24 hr after acute kidney IR. The kidney was collected in each animals for specific study (please see the results of Figure 1).

### Assessment of circulating GLP-1 level and renal function before and after IR procedure

Serum GLP-1, creatinine, blood urea nitrogen (BUN), urine protein, and urine creatinine levels were determined in all animals before and after (at 24 and 72 hours) the IR procedure prior to their sacrifice. Quantification of GLP-1 level, BUN, serum and urine creatinine, and urine protein levels was performed using standard methods according to manufacturers’ instructions.

### Collection of 24-hour urine before and after (on days 1 and 3) IR procedure

For the collection of 24-hr urine for individual study, each animal was put into the animal's metabolic cage [DXL-D, space: 190 × 290 × 550; shu-sz.com, Mainland China] for 24 hrs with food and water supply. Urine in 24 hr was collected in all animals prior to the IR procedure and at 24 hr and 72 hr after reperfusion prior to their sacrifice to determine the daily urine volume and the ratio of urine protein to urine creatinine.

### Histopathology scoring and immunofluorescent (IF) staining at 24 and 72 hr after the IR procedure

Histopathology scoring was determined in a blinded fashion as we previously reported [24]. Briefly, the kidney specimens from all animals were fixed in 10% buffered formalin, embedded in paraffin, sectioned at 5 μm and stained (hematoxylin and eosin; H&E) for light microscopy. The scoring system reflecting the grading of tubular necrosis, loss of brush border, cast formation, and tubular dilatation in 10 randomly chosen, non-overlapping fields (200x) was as follows: 0 (none), 1 (≤10%), 2 (11–25%), 3 (26–45%), 4 (46–75%), and 5 (≥76%) [24].

The IF methodology used in this study have recently been described in details [24]. The IF staining methodology was used for the examination of CD68+ cells (an indicator of macrophage) using respective primary antibodies.

### Western blot analysis of kidney specimens

Equal amounts (10–30 μg) of protein extracts from ischemic kidneys of the animals (n = 10 for each group) were loaded and separated by SDS-PAGE using 7% or 12% acrylamide gradients. The membranes were incubated with monoclonal antibodies against GLP-1R (1:1000, abcam), matrix metalloproteinase (MMP)-9 (1:1000, Millipore), intercellular adhesion molecule (ICAM)-1 (1: 2000, Abcam), NAD(P)H quinone oxidoreductase (NQO) 1 (1: 1000, Abcam), heme oxygenase (HO)-1 (1: 250, Abcam), Glutathione peroxidase (GPx) (1:2000, abcam), and polyclonal antibodies against tumor necrosis factor (TNF)-α (1: 1000, Cell Signaling), nuclear factor (NF)-κB (1:600, Abcam), ADPH oxidase (NOX)-1 (1:1500, Sigma), NOX-2 (1:500, Sigma), Bax (1: 1000, Abcam), caspase 3 (1: 1000, Cell Signaling), poly(ADP-ribose) polymerase (PARP) (1: 1000, Cell Signaling), Bcl-2 (1:250, Abcam), catalase (1:1000, abcam), superoxide dismutase 1 (SOD-1) (1:2000, abcam), γH2AX (1:1000, Cell signaling), and endothelial nitric oxide synthase (eNOS) (1:1000, Abcam) were used. Signals were detected with horseradish peroxidase (HRP)-conjugated goat anti-mouse, goat anti-rat, or goat anti-rabbit IgG.

The Oxyblot Oxidized Protein Detection Kit was purchased from Chemicon (S7150). The procedure of 2,4-dinitrophenylhydrazine (DNPH) derivatization was carried out on 6 μg of protein for 15 minutes according to the manufacturer’s instructions. One-dimensional electrophoresis was carried out on 12% SDS/polyacrylamide gel after DNPH derivatization. Proteins were transferred to nitrocellulose membranes which were then incubated in the primary antibody solution (anti-DNP 1: 150) for two hours, followed by incubation with the second antibody solution (1:300) for one hour at room temperature. The washing procedure was repeated eight times within 40 minutes.

Immunoreactive bands were visualized by enhanced chemiluminescence (ECL; Amersham Biosciences), which was then exposed to Biomax L film (Kodak). For quantification, ECL signals were digitized using Labwork software (UVP). For oxyblot protein analysis, a standard control was loaded on each gel.

### Real-time quantitative PCR analysis

The mRNA expressions of TNF-α, interleukin (IL)-1β, MMP-9, plasminogen activator inhibitor (PAI), IL-10, and endothelial nitric oxide synthase (eNOS) in each of the four groups of animals were analyzed with RT-qPCR and compared.

### Statistical analysis

Quantitative data are expressed as means ± SD. Statistical analyses were performed using SAS statistical software for Windows version 8.2 (SAS institute, Cary, NC) to conduct ANOVA followed by Bonferroni multiple-comparison post hoc test. A probability value <0.05 was considered statistically significant.

## Results

### Exendin-9-39 inhibited the effect of sitagliptin on attenuating the acute kidney IR injury (Figure 1)

**Figure 2:**
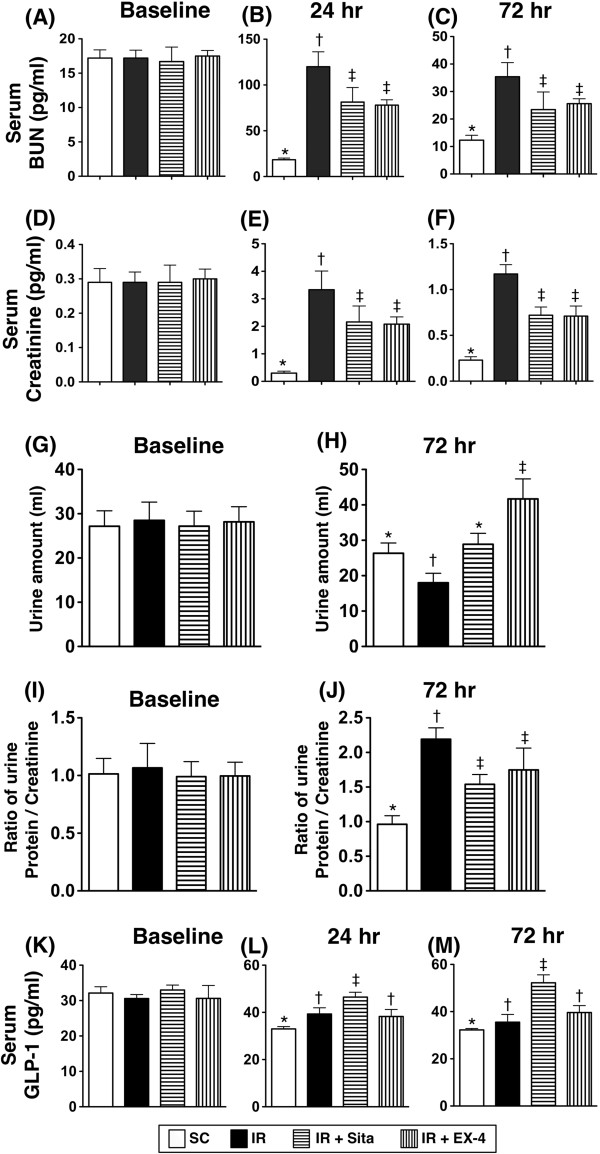
**Serum levels of blood urea nitrogen (BUN), creatinine and glucagon-like peptide-1 (GLP-1), urine amount and ratio of urine protein to creatinine after ischemia-reperfusion (IR) procedure (n = 6 at each time for each group). A)** Baseline serum BUN level; p value >0.5 among the four groups. **B)** Serum level of BUN at 24 h after IR procedure; * vs. other groups with different symbols, p < 0.001. **C)** Serum level of BUN at 72 h after IR procedure; * vs. other groups with different symbols, p < 0.001. **D)** Baseline serum creatinine level; p value >0.5 among the four groups. **E)** Serum level of creatinine at 24 h after IR procedure; * vs. other groups with different symbols, p < 0.001. **F)** Serum level of creatinine at 72 h after IR procedure; * vs. other groups with different symbols, p < 0.001. **G)** Baseline urine amount; p value >0.5 among the four groups. **H)** Urine amount at 72 h; * vs. other groups with different symbols, p < 0.001. **I)** Baseline ratio of urine protein to creatinine; p value >0.5 among the four groups. **J)** Ratio of urine protein to creatinine at 27 after IR procedure; * vs. other groups with different symbols, p < 0.005. **K)** Baseline serum concentration of GLP-1; p value >0.5 among the four groups. **L)** Serum concentration of GLP-1 at 24 h after IR procedure; * vs. other groups with different symbols, p < 0.005. **M)** Serum concentration of GLP-1 at 72 h after IR procedure; * vs. other groups with different symbols, p < 0.001. Statistical analysis using one-way ANOVA, followed by Bonferroni multiple comparison post hoc test (n = 6). Symbols (*, †, ‡) indicate significant difference (< 0.05). SC = sham control; IR = ischemia-reperfusion; Sita = sitagliptin; Ex-4 = extendin-4.

To assess the effect of sitagliptin therapy on ameliorating acute kidney IR was inhibited by extendin-9-39, an antagonist of exendin-4, 24 hr acute kidney IR injury was done in additional six animals, i.e., IR only (n = 2), IR + sitagliptin (n = 2), and IR + sitagliptin + exendin-9-39. The H. & E. stain showed that as compared with IR only, sitagliptin therapy markedly reduced the kidney injury score. However, this treatment effect was notably reduced by extendin-9-39. Additionally, the expression of GLP-1R in kidney parenchyma was notably higher in sitagliptin-treated animals than in those of IR only animals. However, the treatment effect was remarkably diminished by extendin-9-39 treatment. Moreover, the protein expressions of oxidative stress (oxidized protein), ROS (NOX-1, NOX-2), and inflammatory biomarkers (ICMA-1, TNF-α, NF-κB, MMP-9) were markedly lower in sitagliptin-treated animals than in IR only animals. However, despite of the sitagliptin treatment, these protein expressions were up-regulated again by extendin-9-39 therapy in the acute kidney IR animals. Furthermore, after acute kidney IR injury, the circulating level of GLP-1 was significantly higher animals than in other groups of the animals (Figure 2-L, 2-M). Accordingly, our findings supported that the effect of sitagliptin therapy on attenuating acute kidney IR injury was mainly through regulating the circulating level of GLP-1, a signaling pathway similar to exedinin-4.

### Changes in renal functions and circulating levels of GLP-1 at 24 h and 72 h after acute renal IR injury (Figure 2)

Prior to the IR induction, the serum levels of BUN and creatinine were similar among the sham controls (group 1), animals with IR injury only (group 2), IR injury + sitagliptin (group 3), and IR injury + exendin-4 (group 4). However, at 24 hr after reperfusion, the serum levels of BUN and creatinine were significantly higher in group 2 than those in other groups and significantly higher in groups 3 and 4 than those in group 1, but it showed no difference between groups 3 and 4. In addition, at 72 hr after IR procedure, these two parameters showed an identical pattern compared to that of 24 hr among the four groups.

The daily urine amount and the ratio of urine protein to urine creatinine prior to the IR procedure did not differ among the four groups. However, the daily urine amount was significantly less in group 2 than that in other groups and significantly less in group 1 than groups 3 and 4, and significantly less in group 3 as compared to that of the group 4 at 72 hr after reperfusion.

### Histopathological scoring of the kidneys at 24 h and 72 after IR injury (Figure 3)

**Figure 3:**
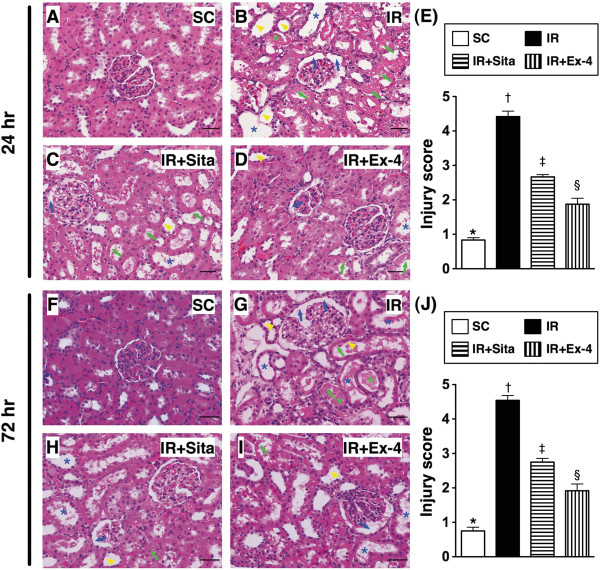
**Histopathological scoring of kidney injury at 24 h and 72 h after ischemia-reperfusion (IR) procedure (n = 6 at each time for each group). A** to **D)** and **F** to **I) H**. &**E**. stain (200 x) showing significantly higher degree of brush border in renal tubules (yellow arrowheads), cast formation (green asterisk), tubular dilatation (blue asterisk), tubular necrosis (green arrows), and dilatation of Bowman’s capsule (blue arrows) in IR only group than in other groups at 24 h **(A to D)** and 72 h **(F to I)** after IR procedure, respectively. **E)** by 24 h after IR procedure: * vs. other groups with different symbols (*, †, ‡, §), p < 0.0001. **J)** by 72 h after IR procedure: * vs. other groups with different symbols (*, †, ‡, §), p < 0.0001. All statistical analyses using one-way ANOVA, followed by Bonferroni multiple comparison post hoc test (n = 6). Symbols (*, †, ‡, §) indicate significance (at 0.05 level). SC = sham control; IR = ischemia-reperfusion; Sita = sitagliptin; Ex-4 = extendin-4.

To evaluate the therapeutic impact of sitagliptin and exendin-4 on IR-induced renal injury, histological scoring based on the typical microscopic features of acute tubular damage, including extensive tubular necrosis and dilatation, as well as cast formation and loss of brush border was adopted. The injury was found to be significantly higher in group 2 than in other groups, significantly higher in groups 3 and 4 than in group 1 (Figure 3A-E), and significantly higher in group 3 than group 4 at 24 h or 72 h after IR procedure (Figure 3F-J). These pathological findings might suggest that on dose of exendin-4 was not inferior to sitagliptin therapy for protecting acute kidney IR injury.

### Changes in mRNA expression of inflammatory and anti-inflammatory biomarkers in renal parenchyma at 72 h after IR injury (Figure 4)

**Figure 4:**
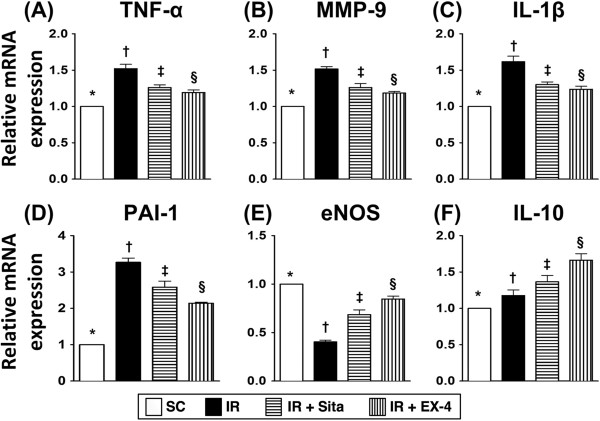
**The gene expressions of inflammatory and anti-inflammatory biomarkers at 72 h after ischemia-reperfusion (IR) procedure (n = 6 at each time for each group). A** to **C)** The mRNA expressions of tumor necrotic factor (TNF)-α **(A)**, matrix metalloproteinase (MMP)-9 **(B)** and interleukin (IL)-β **(C)**; * vs. other groups with different symbols (*, †, ‡, §), p < 0.001. **D)** The mRNA expression of plasminogen activator inhibitor (PAI)-1; * vs. other groups with different symbols (*, †, ‡, §), p < 0.0001. **E** to **F)** The mRNA expressions of endothelial nitric oxide synthase (eNOS) and IL-10; * vs. other groups with different symbols (*, †, ‡, §), p < 0.001. All statistical analyses using one-way ANOVA, followed by Bonferroni multiple comparison post hoc test (n = 6). Symbols (*, †, ‡, §) indicate significance (at 0.05 level). SC = sham control; IR = ischemia-reperfusion; Sita = sitagliptin; Ex-4 = extendin-4.

The mRNA expressions of TNF-1α, MMP-9, and IL-1β, three indicators of inflammation, were remarkably higher in group 2 than those in other groups and significantly higher in groups 3 and 4 than those in group 1, but it showed no difference between group 3 and group 4 (Figure 4A-C). Moreover, the mRNA expression of PAI-1, another indicator of inflammation, was highest in group 2 and lowest in group 1, and significantly higher in group 3 than that in group 4 (Figure 4D). On the other hand, the mRNA expressions of eNOS and IL-10, two anti-inflammatory indexes, were highest in group 1 and lowest in group 2, and significantly higher in group 4 than those in group 3 (Figure 4E-F).

### Expression of glucagon-like peptide-1 receptor (GLP-1R) in kidney at 24 hr and 72 hr after reperfusion (Figure 5)

**Figure 5:**
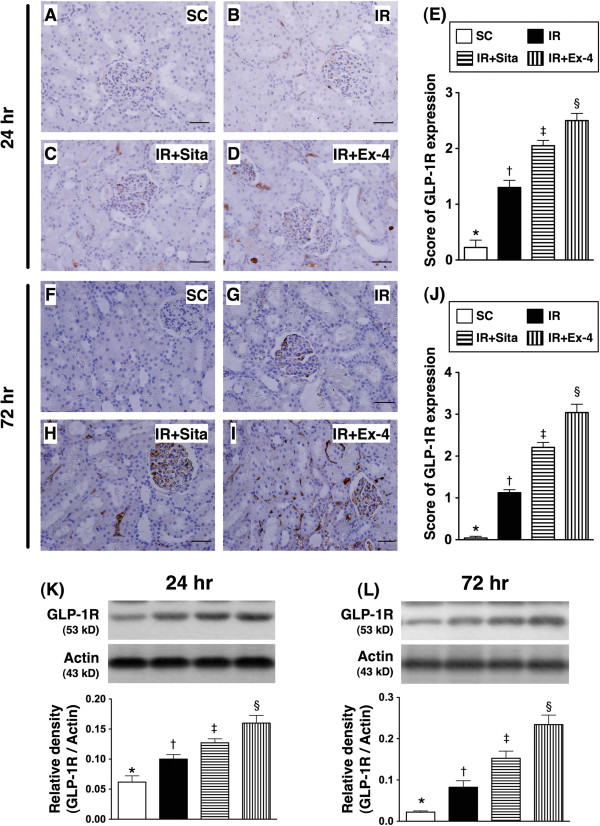
**Immunohistochemical (IHC) staining and Western blot analysis of glucagon-like peptide-1 receptor (GLP-1R) at 24 h and 72 h after ischemia-reperfusion (IR) procedure (n = 6 at each time for each group). A** to **D)** and **F** to **I)** The IHC stain (200 x) identifying the GLP-1R (brown color) in kidney parenchyma among four groups at 24 h **(A to D)** and 72 h **(F to I)** after IR procedure, respectively. **E)** for 24 h after IR procedure: * vs. other groups with different symbols (*, †, ‡, §), p < 0.0001. **J)** for 72 h after IR procedure: * vs. other groups with different symbols (*, †, ‡, §), p < 0.0001. Scale bars in right lower corner represent 50 μm. **K** and **L)** The protein expression of GLP-1R in kidney parenchyma among four group at 24 h **(K)** and 72 h **(L)** after acute IR injury, respectively; for 24 h after IR: * vs. other groups with different symbols (*, †, ‡, §), p < 0.001; for 72 h after IR: * vs. other groups with different symbols (*, †, ‡, §), p < 0.0001. Symbols (*, †, ‡, §) indicate significance (at 0.05 level). Scale bars in right lower corner represent 50 μm. SC = sham control; IR = ischemia-reperfusion; Sita = sitagliptin; Ex-4 = extendin-4.

IHC staining showed that renal GLP-1R expression was highest in group 4 and lowest in group 1, and significantly higher in group 3 than that in group 2 at 24 h (Figure 5A-E) and 72 h (Figure 5F-I) after the procedure. Additionally, the protein expression of GLP-1R in the renal parenchyma showed an identical pattern of IHC staining. These findings suggest that GLP-1R had an intrinsic ability of an auto-regulating expression after acute kidney IR injury and an inversed correlation between the severity of renal IR injury and GLP-1R expression in renal parenchyma (Figure 5K-L).

### Renal infiltration of CD68+ cells at 24 and 72 hr after reperfusion

**Figure 6:**
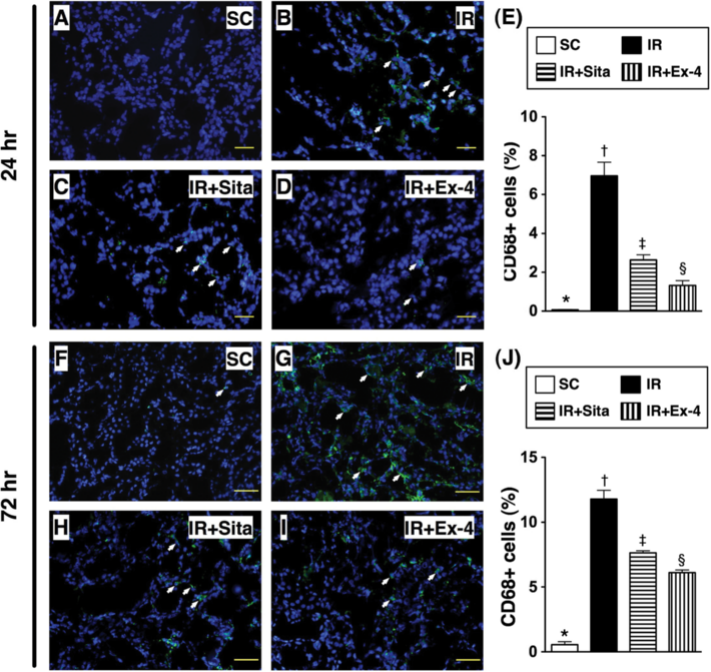
**Immunofluorescent (IF) staining for inflammatory cells (macrophages) at 24 h and 72 after ischemia-reperfusion (IR) procedure (n = 6 at each time for each group). A** to **D)** and **F** to **I)** IF microscopic findings (200 x) showing the number (No.) of CD68+ cell infiltration (white arrows) in kidney parenchyma among four groups at 24 h **(A to D)** and 72 h **(F to I)** after IR procedure, respectively. **E)** The analytical results of CD68+ cells in kidney parenchyma at 24 h after IR procedure. * vs. other groups with different symbols (*, †, ‡, §), p < 0.0001. **J)** The analytical result of CD68+ cells in renal parenchyma at 72 h after IR procedure. * vs. other groups with different symbols (*, †, ‡, §), p < 0.0001. Scale bars in right lower corner represent 50 μm. All statistical analyses were with one-way ANOVA followed by Bonferroni multiple comparison post hoc test. Symbols (*, †, ‡, §) indicate significance at the 0.05 level. SC = sham control; IR = ischemia-reperfusion; Sita = sitagliptin; Ex-4 = extendin-4.

IF staining demonstrated that the number of CD68+ cells (i.e., macrophages), an index of inflammation, was highest in group 2 and lowest in group 1, and significantly higher in group 3 than that in group 4 at 24 hr or 72 hr after reperfusion (Figure 6).

**Figure 7:**
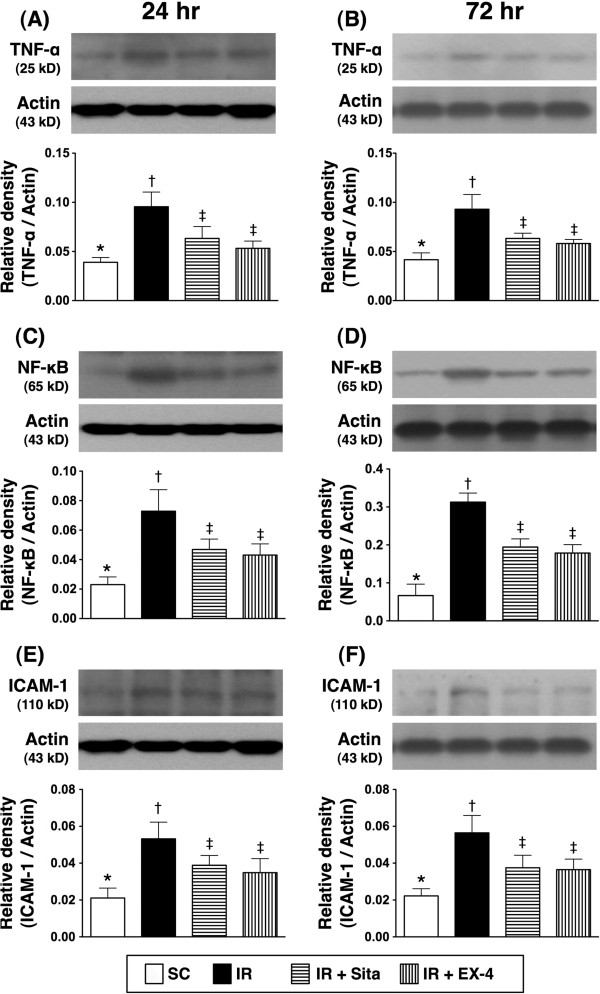
**Changes in protein expressions of tumor necrotic factor (TNF)-α, nuclear factor (NF)-κB and intercellular adhesion molecule (ICAM)-1 in kidney at 24 h and 72 h after ischemia-reperfusion (IR) procedure (n = 6 at each time for each group). A** and **B)** The analytical results of TNF-α protein expression in kidney parenchyma at 24 h **(A)** and 72 h **(B)** after IR procedure. * vs. other groups with different symbols (*, †, ‡), p < 0.01 at 24 h and 72 h, respectively. **C** and **D)** The analytical results of NF-κB protein expression in kidney parenchyma at 24 h **(C)** and 72 h **(D)** after IR procedure. * vs. other groups with different symbols (*, †, ‡), p < 0.001 at 24 h and 72 h, respectively. **E** and **F)** The analytical results of ICAM-1 protein expression in kidney parenchyma at 24 h **(E)** and 72 h **(F)** after IR procedure. * vs. other groups with different symbols (*, †, ‡), p < 0.01 at 24 h and 72 h, respectively. All statistical analyses were with one-way ANOVA followed by Bonferroni multiple comparison post hoc test. Symbols (*, †, ‡) indicate significance at the 0.05 level. SC = sham control; IR = ischemia-reperfusion; Sita = sitagliptin; Ex-4 = extendin-4.

**Figure 8:**
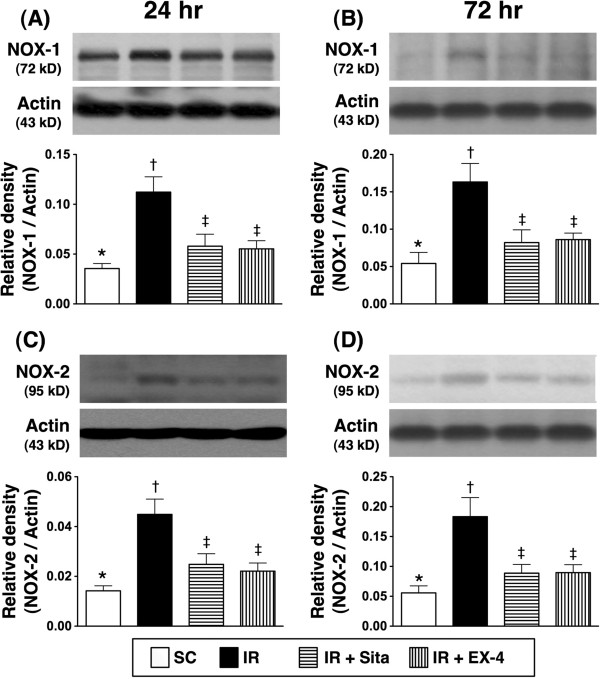
**Changes in protein expressions of NOX-1 and NOX-2 in kidney at 24 h and 72 h after ischemia-reperfusion (IR) procedure (n = 6 at each time for each group). A** and **B)** The analytical results of NOX-1 protein expression in kidney parenchyma at 24 h **(A)** and 72 h **(B)** after IR procedure. * vs. other groups with different symbols (*, †, ‡), p < 0.01 at 24 h and 72 h, respectively. **C** and **D)** The analytical results of NOX-2 protein expression in kidney parenchyma at 24 h **(C)** and 72 h **(D)** after IR procedure. * vs. other groups with different symbols (*, †, ‡), p < 0.01 at 24 h and 72 h, respectively. All statistical analyses were with one-way ANOVA followed by Bonferroni multiple comparison post hoc test. Symbols (*, †, ‡) indicate significance at the 0.05 level. SC = sham control; IR = ischemia-reperfusion; Sita = sitagliptin; Ex-4 = extendin-4.

**Figure 9:**
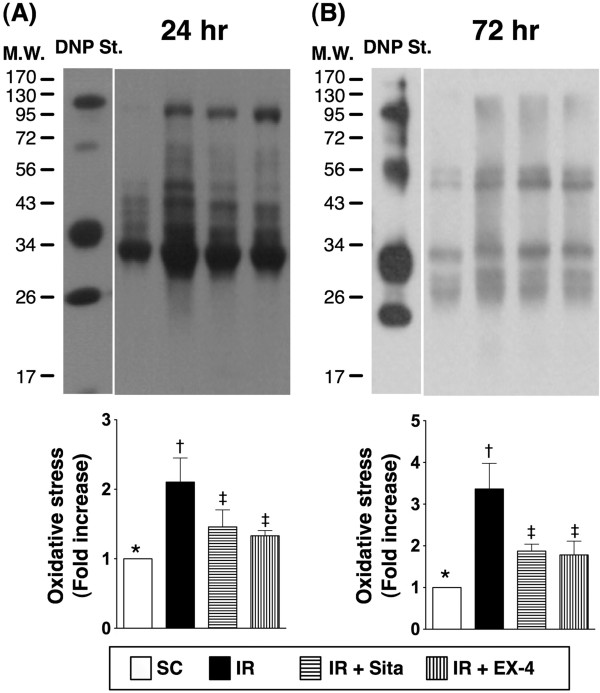
**Changes in protein expressions of oxidative stress in kidney at 24 and 72 h after ischemia-reperfusion (IR) procedure (n = 6 at each time for each group). A** and **B)** Protein expression of oxidative index (protein carbonyls) in kidney parenchyma among four groups of animals at 24 h **(A)** and 48 h **(B)** after IR procedure. * vs. other groups with different symbols (*, †, ‡), p < 0.001 at 24 h and 72 h, respectively. (Note: Right lane and left lane shown on the upper panel represent control oxidized molecular protein standard and protein molecular weight marker, respectively). DNP = 1–3 dinitrophenylhydrazone. All statistical analyses were with one-way ANOVA followed by Bonferroni multiple comparison post hoc test. Symbols (*, †, ‡) indicate significance at the 0.05 level. SC = sham control; IR = ischemia-reperfusion; Sita = sitagliptin; Ex-4 = extendin-4.

The protein expressions of inflammatory, oxidative-stress biomarkers, and reactive oxygen species (ROS) at 24 and 72 hr after IR injury (Figures 7, 8 and 9).

The protein expressions of TNF-α, NF-κB, and ICAM-1, three indicators of inflammation, were significantly higher in group 2 than those in other groups, significantly higher in groups 3 and 4 than those in group 1 at both 24 h and 72 h after IR procedure. No significant difference in the expressions of the three parameters, however, was noted between group 3 and group 4 (Figure 7). Besides, the protein expressions of NOX-1 and NOX-2, two indices of ROS, exhibited an identical pattern compared to that of inflammatory biomarker expressions (Figure 8) among the four groups at the two time points. Furthermore, the expression of oxidized protein, an index of oxidative stress, displayed a pattern similar to that of ROS among the four groups at the two time points (Figure 9).

### The protein expressions of apoptotic, anti-apoptotic, and DNA damage markers at 24 and 72 hr after reperfusion (Figures 10 and 11)

**Figure 10:**
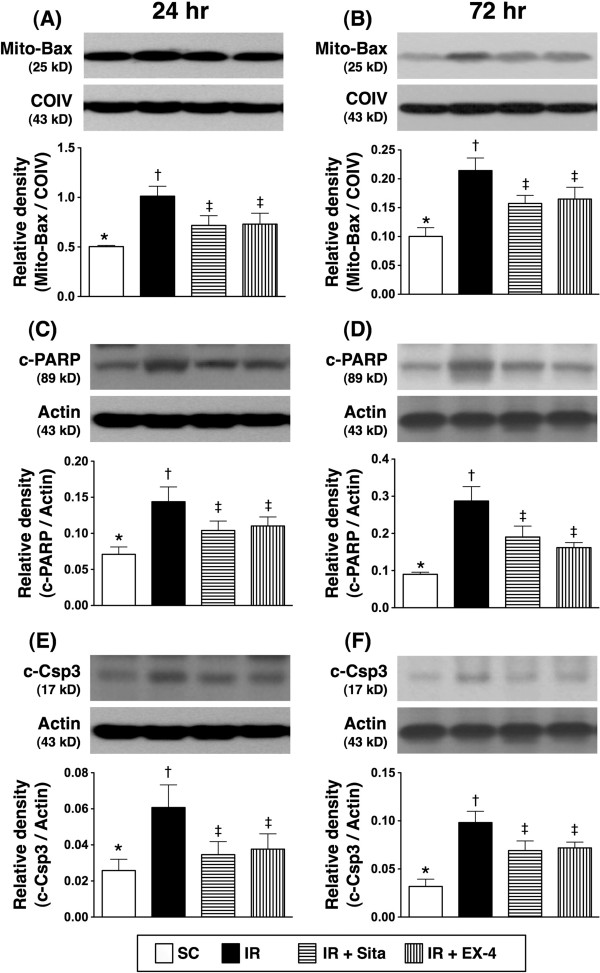
**Changes in protein expressions of apoptotic biomarkers in kidney at 24 and 72 h after ischemia-reperfusion (IR) procedure (n = 6 at each time for each group). A** and **B)** Protein expression of mitochondrial (Mito) Bax at 24 h **(A)** and 72 h **(B)** after acute IR procedure. * vs. other groups with different symbols (*, †, ‡), p < 0.01 at 24 h and p < 0.001 at 72 h, respectively. **C** and **D)** Protein expression of cleaved poly(ADP-ribose) polymerase (c-PARP) at 24 h **(C)** and 72 h **(D)** after acute IR procedure. * vs. other groups with different symbols (*, †, ‡), p < 0.01 at 24 h and p < 0.001 at 72 h, respectively. **E** and **F)** Protein expression of cleaved caspase (c-Csp3) 3 at 24 h **(E)** and 72 h **(D)** after acute IR procedure. * vs. other groups with different symbols (*, †, ‡), p < 0.01 at 24 h and p < 0.001 at 72 h, respectively. All statistical analyses were with one-way ANOVA followed by Bonferroni multiple comparison post hoc test. Symbols (*, †, ‡) indicate significance at the 0.05 level. SC = sham control; IR = ischemia-reperfusion; Sita = sitagliptin; Ex-4 = extendin-4.

**Figure 11:**
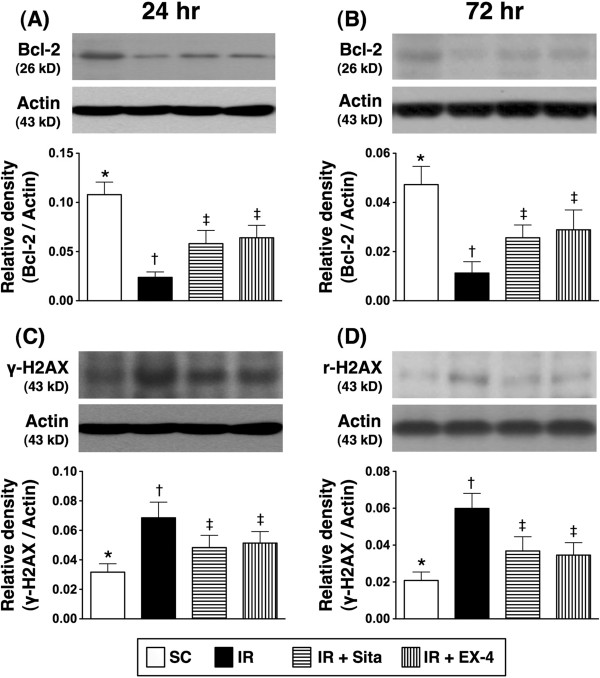
**Changes in protein expressions of Bcl-2 and γH2AX in kidney at 24 and 72 h after ischemia-reperfusion (IR) procedure (n = 6 at each time for each group). A** and **B)** The protein expression of Bcl-2 at 24 h **(A)** and 72 h **(B)** after acute IR procedure. * vs. other groups with different symbols (*, †, ‡), p < 0.001 at 24 h and 72 h, respectively. **C** and **D)** The protein expression of γH2AX at 24 h **(C)** and 72 h **(D)** after acute IR procedure. * vs. other groups with different symbols (*, †, ‡), p < 0.01 at 24 h and p < 0.001 at 72 h, respectively. All statistical analyses were with one-way ANOVA followed by Bonferroni multiple comparison post hoc test. Symbols (*, †, ‡) indicate significance at the 0.05 level. SC = sham control; IR = ischemia-reperfusion; Sita = sitagliptin; Ex-4 = extendin-4.

The protein expressions of mitochondrial Bax and cleaved (i.e., active form) caspase 3 and PARP, three indices of apoptosis, were significantly higher in group 2 than those in other groups, and significantly higher in groups 3 and 4 than those in group 1, but it showed no difference between groups 3 and 4 at 24 hr and 72 hr after reperfusion (Figure 10A-F). Conversely, the protein expression of Bcl-2 showed an opposite pattern compared to that of apoptotic biomarkers after the two intervals of reperfusion (Figure 11). Furthermore, the protein expression of γ-H2AX, an indicator of DNA damage, was significantly higher in group 2 than that in other groups, and significantly higher in groups 3 and 4 than that in group 1, but no difference was noted between groups 3 and 4 at these two time points (Figure 11A-D).

### The protein expressions of anti-oxidative and anti-inflammatory biomarkers at 24 and 72 hr after reperfusion (Figures 12 and 13)

**Figure 12:**
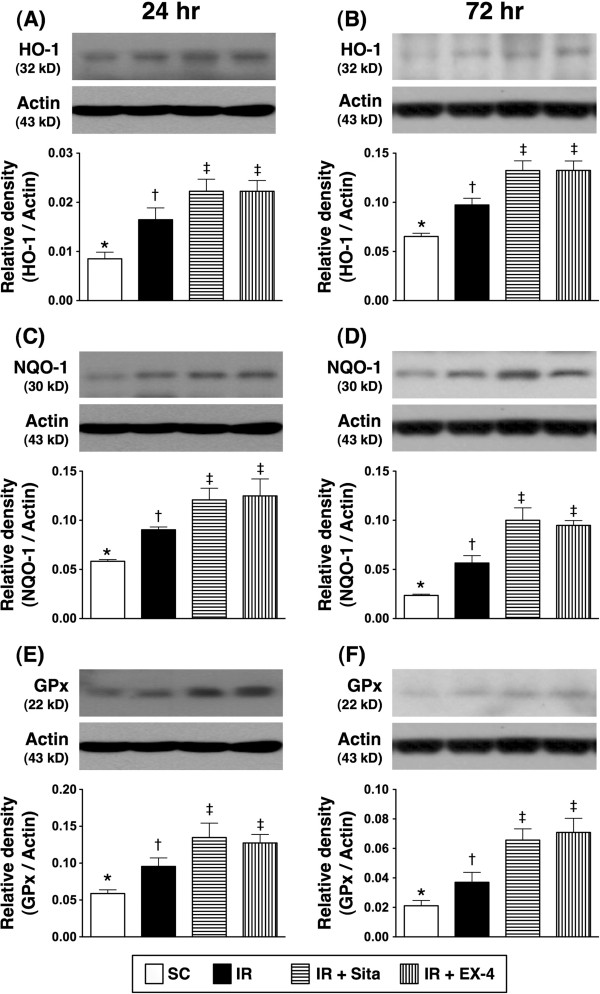
**Changes in protein expressions of anti-oxidant biomarkers in kidney at 24 and 72 h after ischemia-reperfusion (IR) procedure (n = 6 at each time for each group). A** and **B)** The protein expression of heme oxygenase (HO)-1 at 24 h **(A)** and 72 h **(B)** after acute IR procedure. * vs. other groups with different symbols (*, †, ‡), p < 0.001 at 24 h and 72 h, respectively. **C** and **D)** The protein expression of NAD(P)H quinone oxidoreductase (NQO)-1 at 24 h **(C)** and 72 h **(D)** after acute IR procedure. * vs. other groups with different symbols (*, †, ‡), p < 0.001 at 24 h and 72 h, respectively. **E** and **F)** The protein expression of Glutathione peroxidase (GPx) at 24 h **(E)** and 72 h **(F)** after acute IR procedure. * vs. other groups with different symbols (*, †, ‡), p < 0.001 at 24 h and 72 h, respectively. All statistical analyses were with one-way ANOVA followed by Bonferroni multiple comparison post hoc test. Symbols (*, †, ‡) indicate significance at the 0.05 level. SC = sham control; IR = ischemia-reperfusion; Sita = sitagliptin; Ex-4 = extendin-4.

**Figure 13:**
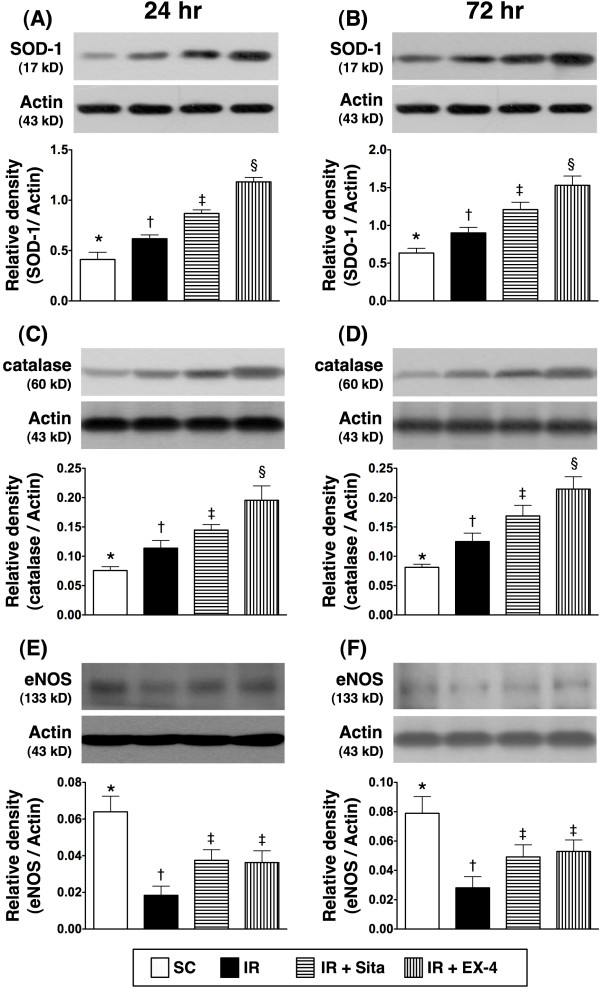
**Changes in protein expressions of anti-superoxide and anti-inflammatory biomarkers in kidney at 24 and 72 h after ischemia-reperfusion (IR) procedure (n = 6 at each time for each group). A** and **B)** The protein expression of superoxide dismutase (SOD)-1 at 24 h **(A)** and 72 h **(B)** after IR procedure. * vs. other groups with different symbols (*, †, ‡), p < 0.001 at 24 h and 72 h, respectively. **C** and **D)** The protein expression of catalse at 24 h **(C)** and 72 h **(D)** after IR procedure. * vs. other groups with different symbols (*, †, ‡), p < 0.001 at 24 h and 72 h, respectively. **E** and **F)** The protein expression of endothelial nitric oxide synthase (eNOS) at 24 h **(E)** and 72 h **(F)** after IR procedure. * vs. other groups with different symbols (*, †, ‡), p < 0.001 at 24 h and 72 h, respectively. All statistical analyses were with one-way ANOVA followed by Bonferroni multiple comparison post hoc test. Symbols (*, †, ‡) indicate significance at the 0.05 level. SC = sham control; IR = ischemia-reperfusion; Sita = sitagliptin; Ex-4 = extendin-4.

The protein expressions of HO-1, NQO-1, and GPx, three indicators of anti-oxidative activities, were not lowest in group 2, and significantly lower in group 1 than that in groups 3 and 4, but it displayed no difference between groups 3 and 4 at 24 h and 72 after IR procedure (Figure 12A-F).

The protein expressions of catalase and SOD-1, two scavengers of superoxide, were lowest in group 1 and highest in group 4, and significantly higher in group 3 than that in group 2 after the two intervals of reperfusion (Figure 13). In addition, the protein expression of eNOS, an indicator of anti-inflammation, was significantly higher in group 1 than that in other groups, significantly higher in groups 3 and 4 than that in group 2, but it showed no difference between groups 3 and 4 after these two time intervals (Figure 13A-F).

## Discussion

The present study, which investigated the therapeutic effect of sitagliptin and exendin-4 against acute renal IR injury, yielded several conspicuous implications.

First, exendin-4 was comparable to sitagliptin in attenuating the architectural integrity of renal parenchyma and arresting the deterioration of renal function after IR injury. Second, either drug remarkably suppressed IR-induced acute kidney injury via inhibiting IR-triggered macrophage recruitment, DNA damage, inflammation, oxidative-stress and ROS generation, as well as through attenuating cellular apoptotic signaling pathway and enhancing GLP-1R expression and anti-oxidant factors in renal parenchyma. Third, to the best of our knowledge, this is the first study to demonstrate the benefits of sitagliptin and exendin-4 in protecting the kidneys from acute IR injury other than their therapeutic actions against hyperglycemia. Of importance is the fact that the results were promising.

### Benefits of sitagliptin and exendin-4 therapy in attenuating IR-induced acute kidney injury---- functional assay and pathological findings

The most distinctive finding in the current study is that the serum BUN and creatinine levels, two important indices of kidney function, were remarkably elevated in animals after acute renal IR injury than those in sham controls. The increases of these parameters were significantly suppressed after sitagliptin or exendin-4 treatment. One important finding is that the ratio of urine protein to creatinine, a useful indicator of impaired renal function, was markedly increased in animals after acute kidney IR compared to that in the sham controls at 24 hr and 72 hr after the procedure. IR-induced elevation of this parameter was significantly suppressed by either sitagliptin or exendin-4 treatment. Another noteworthy finding in the present study is that the histopathological renal injury scores were significantly higher in animals after renal IR than those in sham controls at the two time points, but were significantly reduced by either sitagliptin or exendin-4 therapy. Importantly, this study is the first to demonstrate the therapeutic actions of sitagliptin and exendin-4 in protecting the kidney against acute IR injury other than their roles as hypoglycemic agents. Moreover, the results of the present study also demonstrated comparable protection offered by the two drugs.

### Protection against acute renal IR injury through attenuation of inflammation

Previous studies have shown that ischemia or IR elicits tremendous inflammatory response [20,22,38]. In addition, the initiation and propagation of inflammatory reaction are major contributors to tissue/organ damage after acute IR injury [20,22,38]. One essential finding in the present study is the augmentation the expressions of inflammatory biomarkers at cellular (i.e., macrophage), gene (i.e., MMP-9, IL-1β, TNF-α, PAI-1), and protein (i.e., TNF-α, NF-κB, ICAM-1) levels in kidney parenchyma in the IR animals compared to those in the sham controls not only occurred at 24 hr, but also at 72 hr after reperfusion. Accordingly, our findings are consistent with those of previous studies [20,22,38]. Of importance is the fact that these inflammatory biomarkers were markedly suppressed in the IR animals after receiving sitagliptin or exendin-4 treatment. In this way, our findings further reinforce those of previous studies that also reported the link between the reduction of inflammatory reaction and the preservation of functional integrity of the kidney after ischemia/IR injury [20,38]. Fascinatingly, the expressions of anti-inflammatory biomarkers at gene (IL-10, eNOS) and protein (eNOS) levels were notably enhanced in IR animals after sitagliptin and exendin-4 treatment, highlighting the intrinsic anti-inflammatory properties of the two agents other than their hypoglycemic actions. Therefore, our findings could, at least in part, explain the notably aggravated renal histological distortion and dysfunction in the setting of acute kidney IR and also the mechanisms by which sitagliptin and exendin-4 suppressed the renal IR-induced damage.

### Protection against acute renal IR injury through reduction of oxidative stress

The generation of oxidative stress and ROS have also been shown to play a crucial role in acute kidney IR injury [11,15,20,23-27,39]. The principal finding in the present study is the markedly enhanced protein expressions of oxidative stress (i.e., oxidized protein) and ROS (i.e., NOX-1, NOX-2) in renal parenchyma of animals following acute kidney IR compared to those in the sham controls at both 24 hr and 72 hr after reperfusion. However, the expressions of these biomarkers were notably suppressed in IR animals after receiving either sitagliptin or exendin-4 treatment. Of importance is that the expressions of the anti-oxidative markers at protein level (HO-1, NQO-1, GPx-1, catalase, SOD) was significantly upregulated in the IR animals with either sitagliptin or exendin-4 treatment compared to those without. Beside their well-known roles as hypoglycemic agents, GLP-1 analogues (including exendin-4) have been reported to possess both anti-oxidative properties [29-31] and anti-inflammatory [31-33] properties. Moreover, sitagliptin, an oral hyperglycemic agent, has been found to be capable of enhancing circulating GLP-1 levels via suppressing DPP-IV activity [35,36], thereby contributing to its anti-inflammatory and anti-atherosclerotic cardiovascular protective effect [37]. Our findings, therefore, in addition to being supported by the previous studies [29-37], could further explain the protective effects of sitagliptin and exendin-4 against acute renal IR injury.

### Protection against acute renal ir injury through suppression of cellular apoptosis and DNA damage

Inevitably, cellular apoptosis always takes place after acute ischemia/IR injury [21,38,39]. An association between cellular apoptosis and organ dysfunction has long been identified by experimental studies [21,38,39]. An important finding in the present study is the significantly elevated protein expressions of apoptotic (i.e., cleaved caspase 3 and PARP, mitochondrial Bax) and DNA damage (i.e., γ-H2AX) biomarkers in renal parenchyma of IR animals compared to those in the sham controls at both 24 hr and 72 hr following reperfusion. In this way, our findings corroborated those of previous studies [21,38,39]. However, these biomarkers were substantially reduced in the kidney parenchyma of IR animals after receiving either sitagliptin or exendin-4 treatment. Besides, the protein expression of the anti-apoptotic biomarker, i.e., Bcl-2, was notably augmented after treatment with either agent. Our findings could partially account for the suppressed IR-induced renal histopathological damage after treatment with sitagliptin and extendin-4.

### Protection against acute renal IR injury through enhancing circulating GLP-1 level and GLP-1R expression in renal parenchyma

Although the distribution of GLP-1 binding sites (i.e., GLP-1R) in the central nervous system and the peripheral autonomic nervous system has been extensively investigated in previous studies [40-45], the expression of GLP-1R in renal parenchyma has not been reported. One interesting finding in the current study is the significantly higher circulating GLP-1 level in IR animals with and without exendin-4 treatment than that in the sham controls and also the highest level in IR animals receiving sitagliptin treatment. This may be the result of stress stimulation from IR injury that enhanced the generation of GLP-1 from the digestive system. Additionally, the highest circulating level of GLP-1 after sitagliptin treatment could be due to the inhibitory effect of sitagliptin on the enzymatic activity of DDP-IV which has been found to cleave GLP-1 in the circulation [35,36]. The novel finding in the present study is that, under normal situation, GLP-1 binding sites were rare in the kidney parenchyma as shown in immunohistochemical staining and western blotting. However, during acute kidney IR injury, the expression of GLP-1 binding sites (i.e., through western blot) was markedly enhanced in the kidney parenchyma. The other novel and interesting finding (i.e., IHC staining result) is the predominant distribution of GLP-1 binding sites in the both glomeruli and renal tubules.

Another distinctive finding is that the protein expression of GLP-1 binding sites in kidney parenchyma was rare in normal condition that was only markedly augmented after acute IR injury. Of particularly distinctive finding was that the expression of this biomarker in renal parenchyma was significantly higher in IR animals with sitagliptin treatment than in IR animals without treatment and further significantly higher in IR animals after receiving exendin-4 treatment. These findings suggest an automatic up-regulating expression of GLP-1 binding sites in IR animals after both drug treatment. Of importance is that these findings (i.e., circulating GLP-1 and GLP-1 binding sites in kidney parenchyma) not only were consistent with our hypothesis, but also provided a good positive correlation between the up-regulated expression of GLP-1 binding sites and suppressing the generations of inflammation, oxidative stress, and ROS in the present study.

### Study limitations

**Figure 14:**
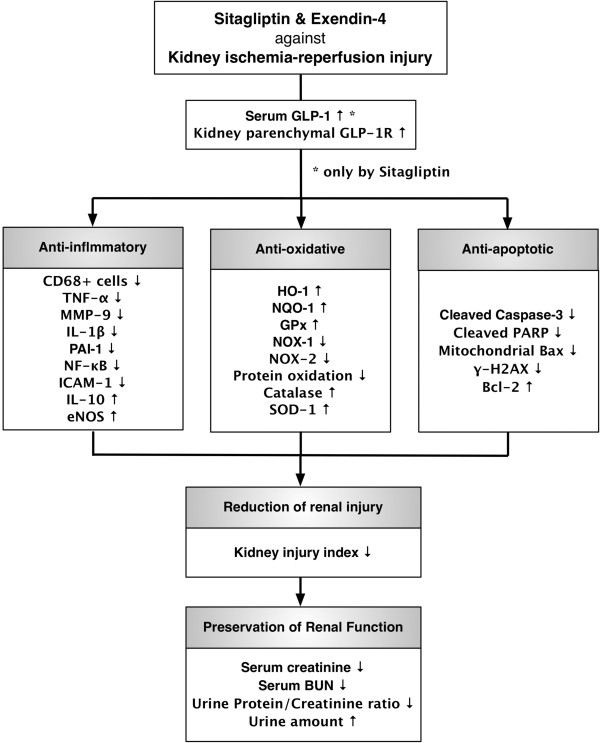
**Proposed mechanisms underlying the positive therapeutic effects of sitagliptin and exendin-4 on kidney ischemia-reperfusion (IR) injury.** GLP-1R = glucagon-like peptide-1 receptor; TNF = tumor necrotic factor; MMP = matrix metalloproteinase; IL = interleukin; PAI = plasminogen activator inhibitor; NF = nuclear factor; ICAM = intercellular adhesion molecule; eNOS = endothelial nitric oxide synthase; HO = heme oxygenase; NQO = NAD(P)H quinone oxidoreductase; GPx = glutathione peroxidase; NOX = ADPH oxidase; SOD = superoxide dismutase; PARP = poly(ADP-ribose) polymerase; BUN = blood urine nitrogen.

This study has several limitations. First, we remain uncertain regarding the explanation of the finding that exendin-4 had relatively higher potency than that of sitagliptin in suppressing kidney injury score and inflammatory cells (CD68+) and in up-regulating the expressions of GLP-1R and anti-oxidants (SOD, catalase). This is perhaps due to the fact that exendin-4, a GLP-1 analogue, possess stronger anti-oxidative [29-31] and anti-inflammatory [31-33] properties compared to those of sitagliptin. Second, despite extensive investigation in the current study, the precise signaling pathway(s) through which sitagliptin and exendin-4 exert their therapeutic effects have not been elucidated. We have, however, proposed the mechanisms based on the findings of the current study as summarized in Figure 14. Third, although the rationale of using sitagliptin and exendin-4 was elucidated in the present study, we did not test the potential toxicity of these two drugs in the setting of acute renal injury. In fact, the dosage of sitagliptin has been recommended to be reduced by half if the patient's estimated glomerular filtration rate (eGFR) is < 30 mL/min/1.73 m^2^. Therefore, the regimen dosage of this study is not recommended to extrapolate to humankind in critical settings such as contrast media-induced nephropathy, shock followed by resuscitation in the emergency and intensive care, kidney transplantation, sepsis or cardiovascular surgery.

In conclusion, acute kidney IR injury significantly augmented GLP-1R expression in kidney parenchyma that were further augmented after sitagliptin or exendin-4 therapy. Either sitagliptin or exendin-4 treatment effectively protected the kidney from IR injury through the suppression of inflammatory reaction, apoptosis, oxidative stress in a rodent model of renal IR injury.

## Competing interests

The authors declare that they have no competing interests of any sort, including commercial association, such as consultancies, stock ownership or other equity interests or patent-licensing arrangements.

## Authors’ contributions

CYT, TTH, and HKY participated in the design of the study, data acquisition and analysis as well as drafting the manuscript. CKS, SL, YCC, and CLT were responsible for the laboratory assay and troubleshooting. LTC, THT,SCK, YLC, CCL, CHH and HWC participated in data acquisition, analysis, and interpretation. ZYY, HWC, SL, and HKY conceived of the study, and participated in its design and coordination and helped to draft the manuscript. All authors read and approved the final manuscript.

## Authors’ information

Yen-Ta Chen and Tzu-Hsien Tsai equal contribute to first authors.

Yung-Lung Chen and Hon-Kan Yip equal contribute to corresponding authors.
